# Supplementary material for: Snord67 promotes breast cancer metastasis by guiding U6 modification and modulating the splicing landscape
Source: Nat Commun. 2025 May 2;16:4118. doi: 10.1038/s41467-025-59406-w (PMC12048515; doi:10.1038/s41467-025-59406-w)
Supplement: Supplementary file 1 — Supplementary Information [file 41467_2025_59406_MOESM1_ESM.pdf]

Snord67 promotes breast cancer metastasis by guiding U6 modification and modulating the splicing landscape

Yvonne L. Chao, Katherine I. Zhou, Kwame K. Forbes, Alessandro Porrello, Gabrielle M. Gentile, Yinzhou Zhu, Aaron C. Chack, Dixcy J.S. John Mary, Haizhou Liu, Eric Cockman, Lincy Edatt, Grant A. Goda, Justin J. Zhao, Hala Abou Assi, Hannah J. Wiedner, Yihsuan Tsai, Lily Wilkinson, Amanda E.D. Van Swearingen, Lisa A. Carey, Jimena Giudice, Daniel Dominguez, Christopher L. Holley, Chad V. Pecot

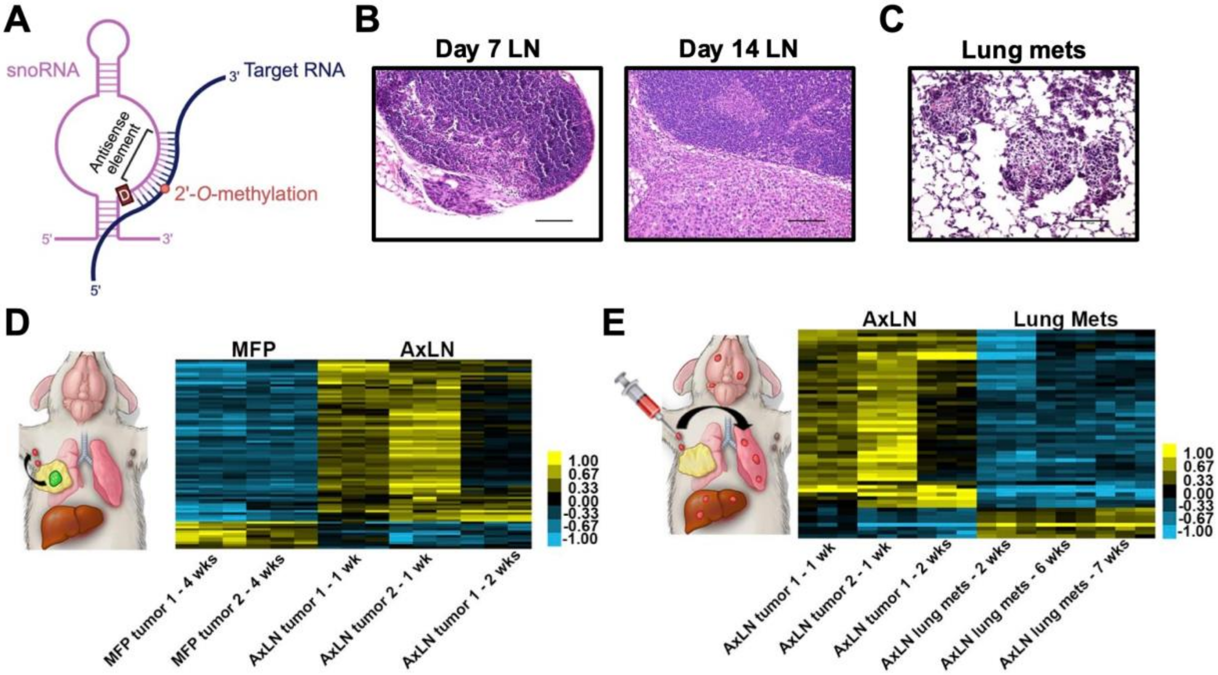

Supplemental Figure 1. Identification of snoRNAs among ncRNAs and poorly characterized RNAs that are differentially expressed in axillary LN tumors by microarray profiling. (**A**) Schematic illustrating the structure of a snoRNA and its interaction with a target RNA. Created in BioRender. Zhou, K. (2025) https://BioRender.com/m67e606. (**B**) H&E-stained images of axillary lymph node tumors at 7 and 14 days after micro-injection of 4T1 cells. Scale bar = 100 μm. (**C**) H&E-stained images of lung metastases derived from micro-injected axillary lymph node tumors. Scale bar = 100 μm. (**D**) Pairwise comparison of differentially expressed ncRNAs in AxLN tumors (n=3) vs. MFP tumors (n=2). (**E**) Pairwise comparison of differentially expressed ncRNAs in lung metastases derived from AxLN tumors (n=3) vs. AxLN tumors (n=3). Tumors were harvested at the indicated timepoints and expanded *ex vivo* to generate subclones. n=3 biologic replicates. For (D) and (E), expression values were plotted on the heat maps using colors (yellow-blue) assigned by the TreeView plotting software, with the contrast set to 1 (see the color bar). Mouse graphics: © 2020 The University of Texas M.D. Anderson Cancer Center.

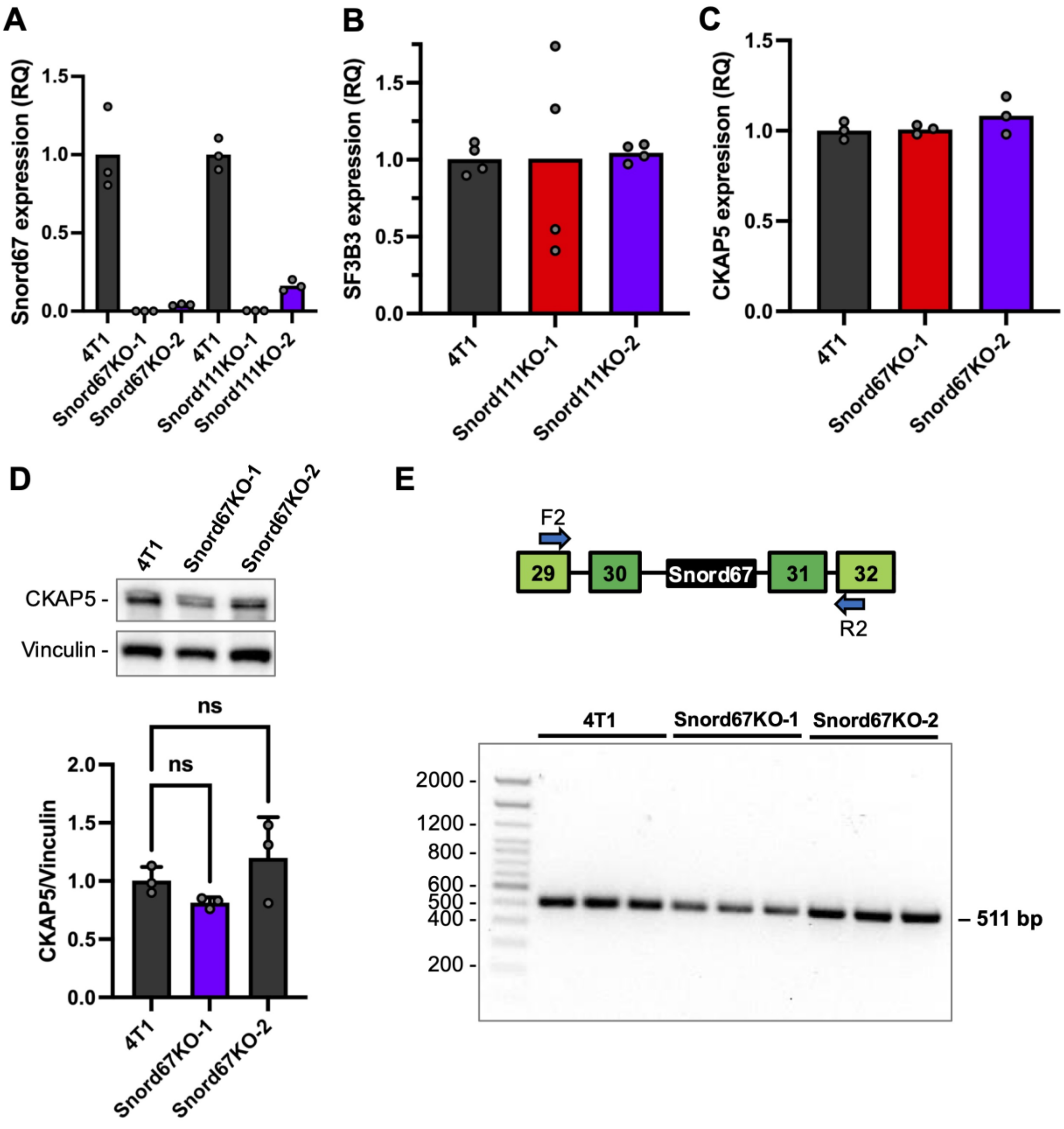

Supplemental Figure 2. Snord67 knockout does not affect host gene expression in 4T1. (A) Expression of Snord67 and Snord111 in 4T1 WT cells and CRISPR knockout cells as quantified by qPCR and presented as relative quantification (RQ) compared to 4T1 WT. n=3 technical replicates. (**B–C**) Expression of Snord67 host gene *CKAP5* and Snord111 host gene *SF3B3* as quantified by qPCR (primers: Ckap5 F1 and Ckap5 R1; Sf3b3 F and Sf3b3 R), presented as RQ compared to 4T1 WT. n=3 (B) or 4 (D) technical replicates. (**D**) Western blot of CKAP5 and vinculin in 4T1 WT cells and two Snord67 knockout clones, p=0.49 (4T1 vs. Snord67KO-1), 0.47 (4T1 vs. Snord67KO-2) by one-way ANOVA. (**E**) RT-PCR of CKAP5 using primer Ckap5 F2 (in exon 29) and primer Ckap5 R2 (in exon 32) to test for alternative splicing of the intron containing Snord67 or alternative splicing of exons 30–31 flanking this intron. n=3 biological replicates, error bars = +1 S.D.

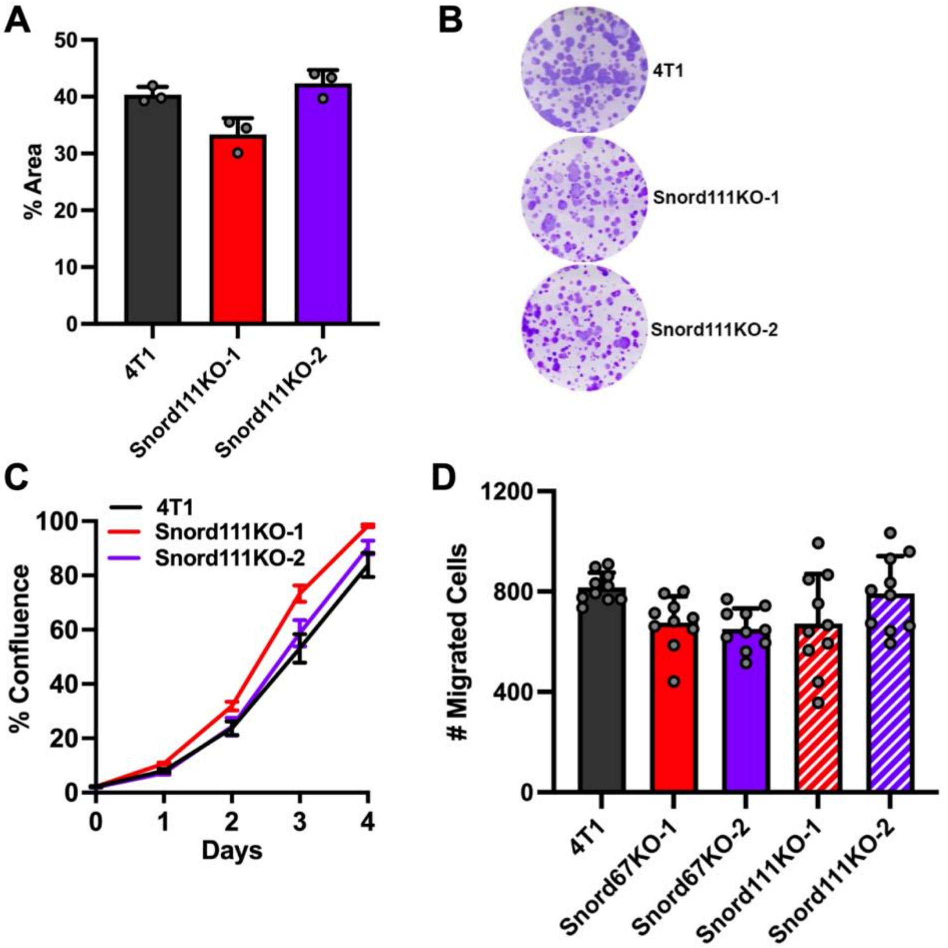

Supplemental Figure 3. Snord111 knockout does not affect colony formation or cell proliferation, and Snord67 knockout does not affect migration. (**A–B**) Colony formation assay demonstrating tumorigenesis. 4T1 WT cells and Snord111KO cells were plated in triplicate, and colonies were stained and quantified in relation to the area of each well that was covered after 7 days, error bars = +1 S.D. (**C**) Cell proliferation assay of 4T1 WT cells and Snord111KO cells. Growth of cells was captured as time-lapse images using IncuCyte over 5 days. Growth was quantified as percent confluence of each well. n=4 biologic replicates, error bars = ±1 S.E.M. (**D**) Quantification of migration of 4T1 cells, Snord67KO cells, and Snord111KO cells by trans-well assay. Migration was measured as the number of cells on the underside of the migration chamber membrane after 18 hours, error bars = +1 S.D.

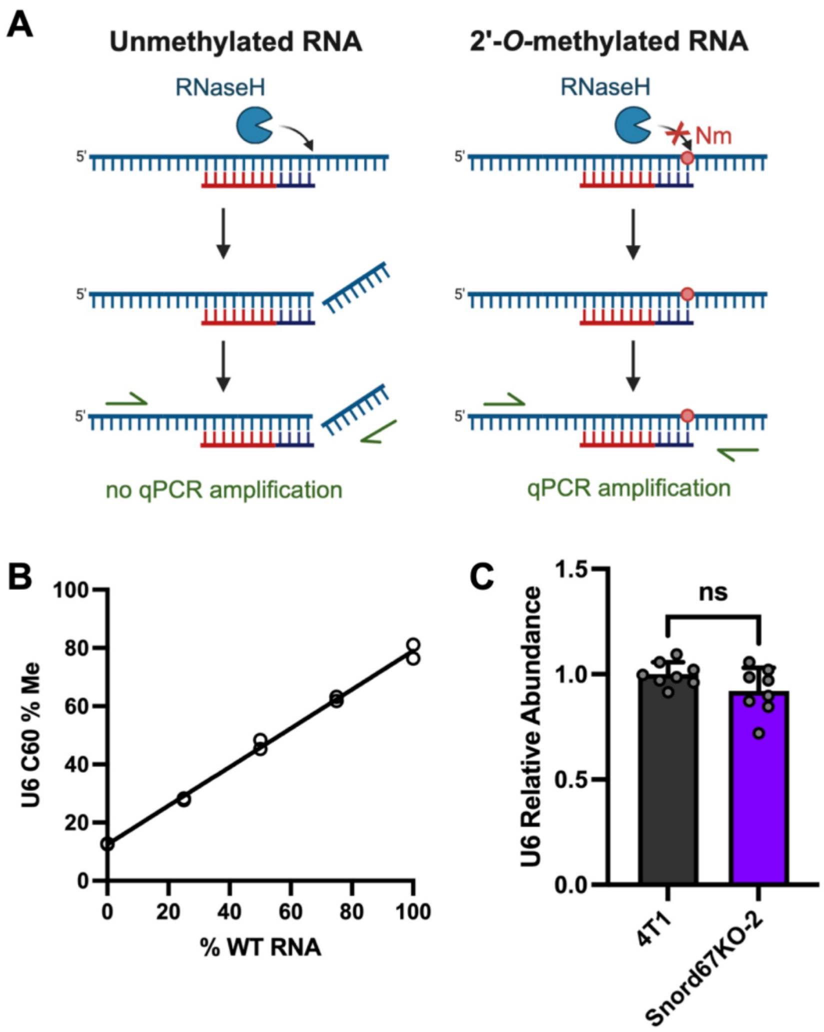

Supplemental Figure 4. Snord67 mutant and site-specific measurement of 2′-*O*-methylation by Nm-VAQ. (**A**) Schematic of the Nm-VAQ method. Chimeras composed of 2′-*O*-methylated (Nm) RNA (red) and DNA (dark blue) are designed to guide RNase H-mediated cleavage of the target RNA (blue) at the 2′-*O*-methylation site of interest. The presence of a 2′-*O*-methylated nucleotide (Nm) at that site blocks cleavage of the target RNA by RNase H. RT–qPCR with flanking primers is then used to measure the fraction of cleaved RNA and to calculate the percent 2’-*O*-methylation at that site. Created in BioRender. Zhou, K. (2025) https://BioRender.com/v61g447. (**B**) Total RNA isolated from EO771.LMB WT cells and from Snord67KO cells were mixed at varying ratios to generate a standard curve. U6 C60 2′-*O*-methylation increases linearly with the percentage of WT RNA in the mixture. (**C**) U6 expression levels in 4T1 WT cells and Snord67KO-2 cells, as measured by RT–qPCR. Error bars correspond to +1 S.D. The difference in U6 expression was not significant (ns) by Student’s t-test (p=0.09).

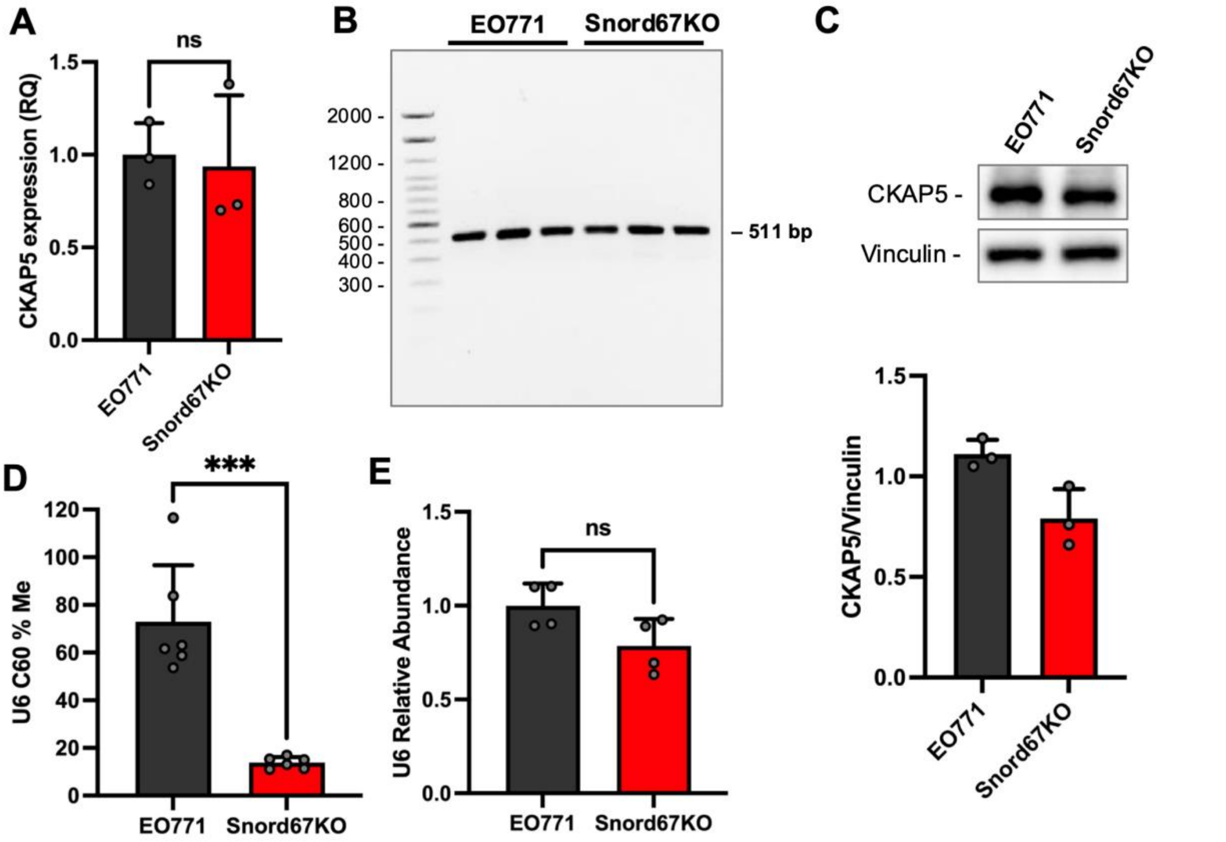

Supplemental Figure 5. Impact of Snord67 knockout on CKAP5, U6, and U6 C60 2′-*O*-methylation in EO771.LMB. (**A**) Expression of CKAP5 in EO771.LMB WT (EO771) and Snord67 knockout (Snord67KO) cells as quantified by RT–qPCR using primers Ckap5 F3 and Ckap5 R3, presented as relative quantification (RQ) normalized to EO771 WT. n=3 biological replicates. Statistical significance was determined by t-test (*p*=0.81). (**B**) RT–PCR of CKAP5 using primers Ckap5 F2 and Ckap5 R2 flanking the intron containing Snord67. n=3 biological replicates. (**C**) Western blot of CKAP5 and vinculin in EO771.LMB WT cells Snord67KO cells. (**D**) U6 C60 methylation levels in EO771 WT cells and Snord67KO cells as measured by Nm-VAQ. Statistical significance was determined by t-test (*p*=0.0001). (**E**) U6 snRNA expression levels in EO771 WT cells and Snord67KO cells as measured by RT–qPCR. Statistical significance was determined by t-test (*p*=0.06). For all bar plots, error bars correspond to +1 S.D. ns = not significant, ***p<0.001.

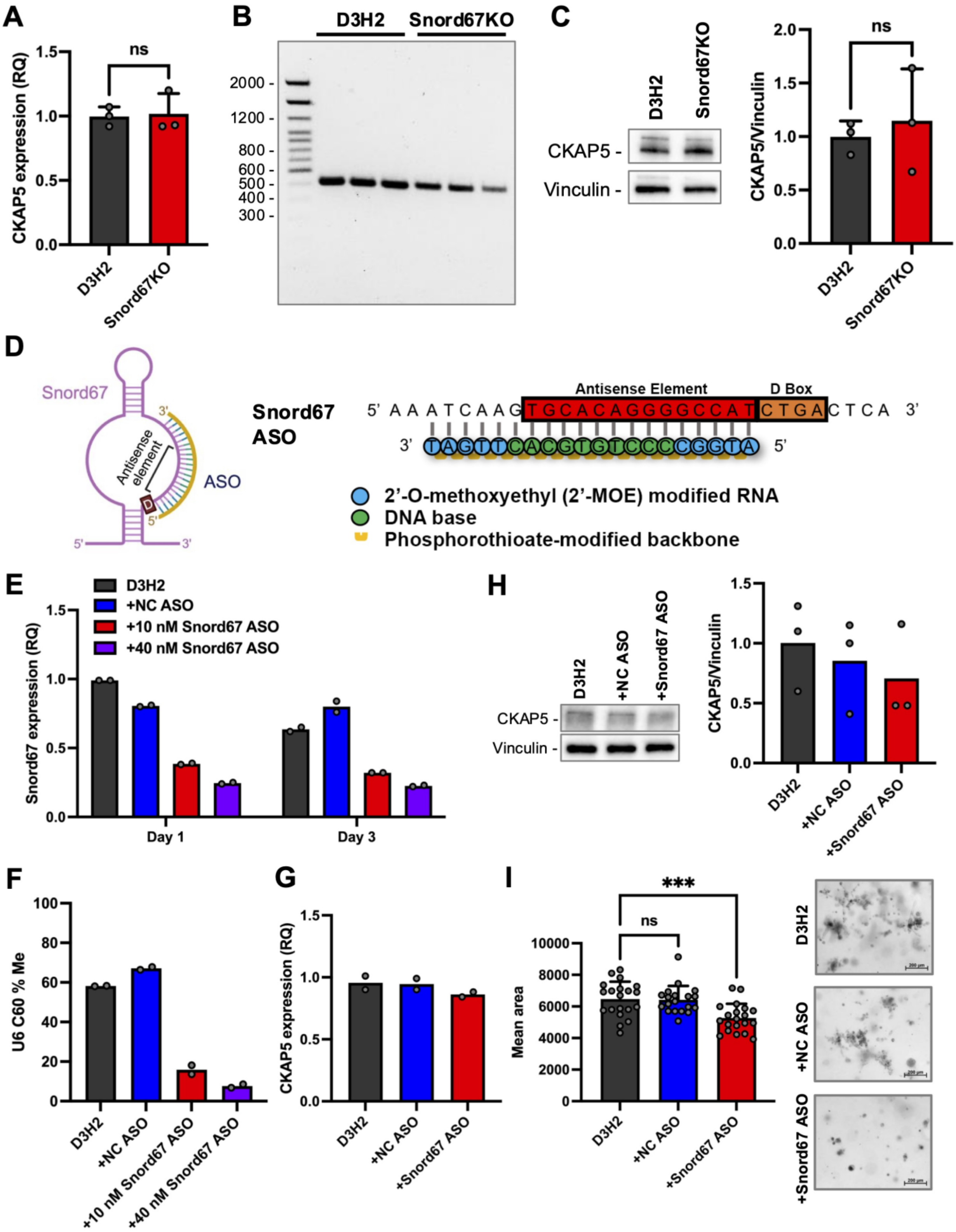

Supplemental Figure 6. Impact of Snord67 knockout and Snord67 ASO on CKAP5 expression in D3H2. (**A**) RQ of CKAP5 in D3H2 WT (D3H2) and Snord67KO cells by RT–qPCR (primers hCkap5 F1 and hCkap5 R1). n=3 biological replicates. *p*=0.85. (**B**) RT–PCR of CKAP5

using primers hCkap5 F2 and hCkap5 R2 flanking the intron containing Snord67. n=3 biological replicates. (**C**) Western blot of CKAP5 and vinculin in EO771.LMB WT cells and Snord67KO cells. p=0.64. (**D**) *Left*: Diagram showing binding of ASO to antisense element of Snord67. Created in BioRender. Zhou, K. (2025) https://BioRender.com/o07w729. *Right*: Design of Snord67 ASO with a phosphorothioate-modified backbone and flanking 2′-methyoxyethyl-modified nucleotides at the 5′ and 3′ ends. (**E**) RQ of Snord67 by RT–qPCR at 1 and 3 days (n=2 technical replicates, normalized to GAPDH, repeated independently 3 times with similar results) and (**F**) U6 C60 methylation levels by Nm-VAQ at 3 days (n=2 technical replicates) after treatment with transfection reagent alone (D3H2) or transfection with 10 nM negative control ASO (+NC ASO) or with 10 or 40 nM Snord67 ASO. (**G**) RQ of CKAP5 at 3 days after treatment with transfection reagent alone (D3H2) or transfection with 40 nM NC or Snord67 ASO by RT–qPCR (primers hCkap5 F1 and hCkap5 R1, n=2 technical replicates). (**H**) Western blot of CKAP5 and vinculin in D3H2 cells with or without transfection of NC or Snord67 ASO (n=3 technical replicates, repeated independently 2 times with similar results). (**I**) Mean spheroid area of D3H2 cells at 7 days after plating in Matrigel and 8 days after treatment with transfection reagent alone (D3H2) or transfection with 40 nM NC or Snord67 ASO, quantified using 19 (+NC ASO) or 20 (D3H2, +Snord67 ASO) images, p=0.99 (D3H2 vs. +NC ASO), 0.0005 (D3H2 vs. +Snord67 ASO). Scale bar = 200 µm. Bar plots show mean, error bars = +1 S.D. Statistical significance was calculated by two-tailed t-test for two-sample comparisons, or by one-way ANOVA for comparisons of more than two samples; ns = not significant, ***p<0.001.

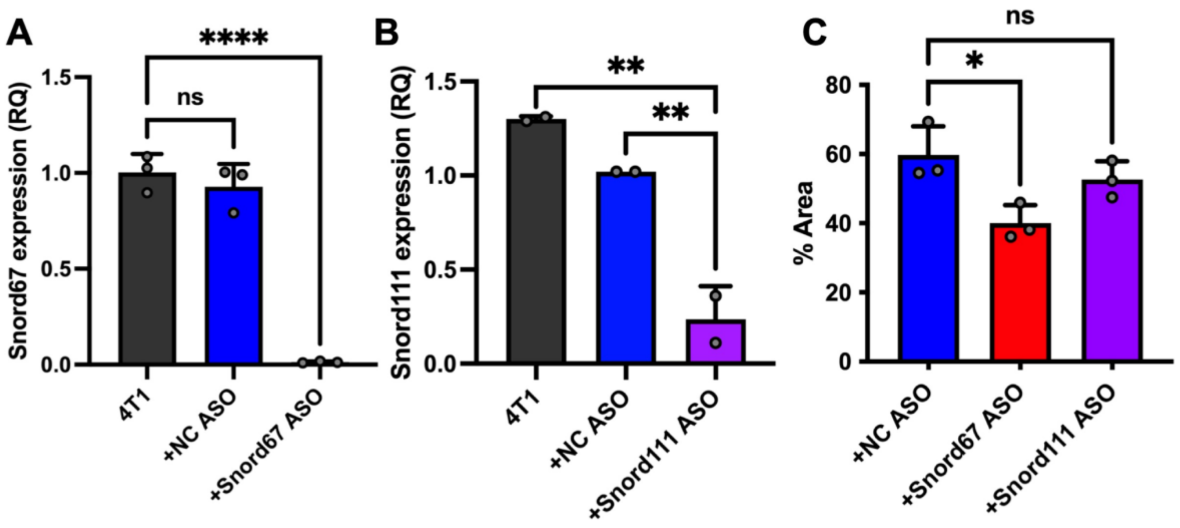

Supplemental Figure 7. (**A**) RT-qPCR relative quantification of Snord67 expression in untreated 4T1 WT cells and in 4T1 WT cells following treatment with 1 μM negative control (NC) or Snord67 ASO for 48 hours *in vitro*. p=0.52 for 4T1 vs. +NC ASO, p<0.001 for 4T1 vs. +Snord67 ASO. (**B**) RQ of Snord111 expression by RT–PCR following treatment of 4T1 WT cells with no ASO, 1 μM NC ASO, or 1 μM Snord111 ASO for 72 hours *in vitro*. p=0.0031 for 4T1 vs. +Snord111 ASO, p=0.0076 for 4T1 vs. +Snord111 ASO. (**C**) 4T1 WT cells were treated with either NC ASO, Snord67 ASO, or Snord111 ASO, and then replated in triplicate for a colony formation assay. After staining, the percentage of each well covered by colonies was quantified. p=0.017 for +NC ASO vs. +Snord67 ASO, p=0.36 for +NC ASO vs. +Snord111 ASO. For all panels, error bars correspond to +1 S.D., and statistical significance was calculated by one-way ANOVA; **p<0.01, ****p<0.0001.

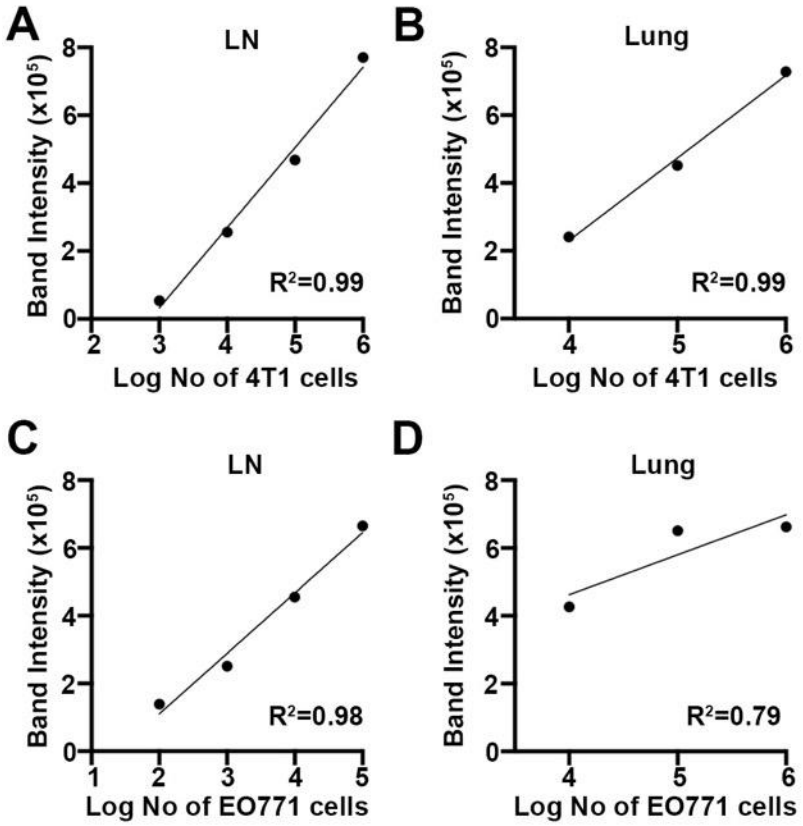

Supplemental Figure 8. Quantification of LN and lung metastases from MFP tumor. Known numbers of 4T1 or EO771.LMB cells were mixed with homogenized LN (**A and C**) or lung (**B and D**) tissues, and then a standard curve was created using qPCR quantification of reporter gene mCherry expression. Due to primer dimers, qPCR amplicons were resolved by agarose gel, and then band intensity was quantified to determine mCherry expression.

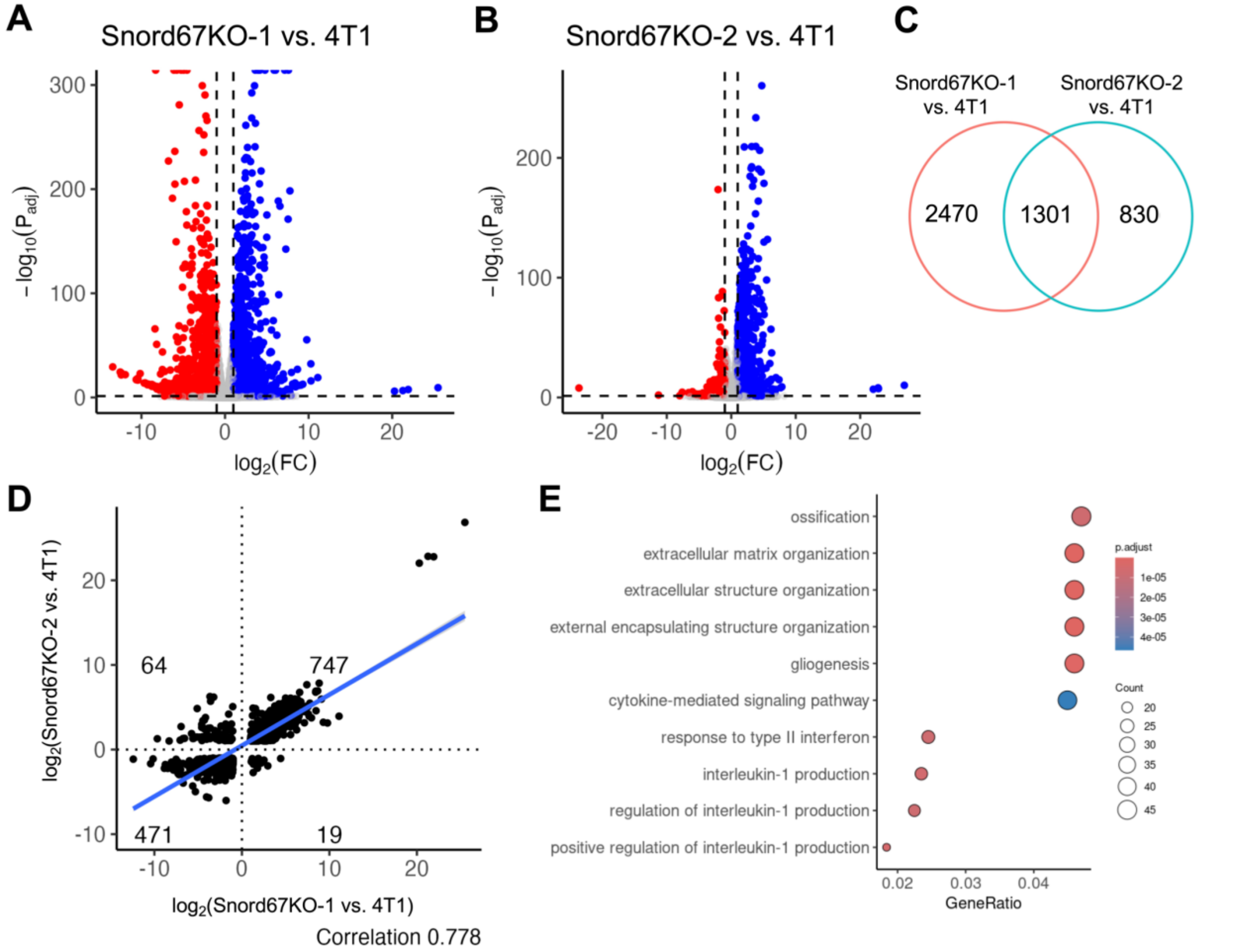

Supplemental Figure 9. Differentially expressed genes in 4T1 WT and Snord67 knockout cells. (**A**) Volcano plot of differentially expressed genes with |log2(foldchange)| > 1 and an adjusted p-value < 0.05 in Snord67KO-1 versus 4T1 WT cells. Genes that are significantly upregulated in Snord67KO-1 cells are shown in blue, and genes that are significantly downregulated are shown in red. (**B)** Volcano plot of differentially expressed genes with |log2(foldchange)| > 1 and an adjusted *p*-value < 0.05 in Snord67KO-2 versus 4T1 WT cells. Significantly upregulated genes are in blue, and significantly downregulated genes in red. (**C**) Venn diagram showing the overlap between differentially expressed genes identified in both Snord67 knockout clones relative to 4T1 WT. (**D**) Correlation between differential gene expression in both Snord67 knockout clones relative to 4T1 WT cells. Numbers designate the number of genes in each quadrant. Spearman's correlation coefficient is shown. (**E**) Gene ontology analysis of 1,301 genes that were differentially expressed in both Snord67 knockout clones relative to 4T1 WT. Enrichment analysis was performed in R using the enrichGO function with a *p*-value cut-off of <0.05, and the Benjamini-Hochberg procedure was applied to adjust for multiple testing.

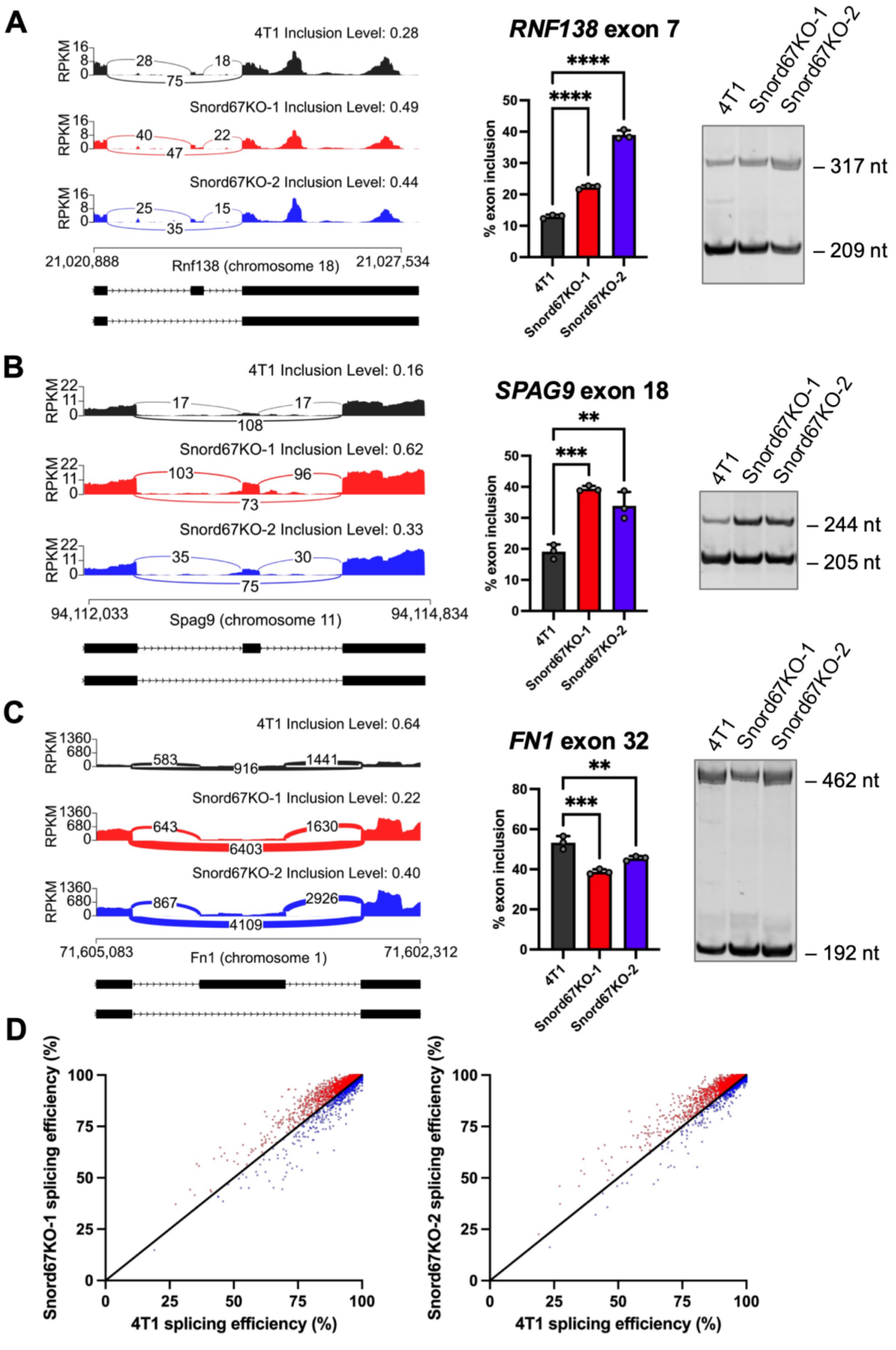

Supplemental Figure 10. Differential alternative splicing and splicing efficiency in Snord67 knockout vs 4T1 WT. Sashimi plots, RT–PCR gels, and quantification of RT–PCR gels for

differentially spliced (**A**) exon 7 in the *RNF138* gene ($p<0.0001$ for both comparisons), (**B**) exon 18 in the *SPAG9* gene ($p=0.0003$ for 4T1 vs. Snord67KO-1, 0.0016 for 4T1 vs. Snord67KO-2), and (**C**) exon 32 in the *FN1* gene ($p=0.0003$ for 4T1 vs. Snord67KO-1, 0.0071 for 4T1 vs. Snord67KO-2). The RNA-seq and RT–PCR experiments were performed in n=3 biological replicates. Error bars correspond to +1 S.D. Statistical significance was determined by one-way ANOVA; $^{**}p<0.01$, $^{***}p<0.001$, $^{****}p<0.0001$. (**D**) Splicing efficiency of each intron in Snord67KO-1 cells vs. 4T1 WT cells (*left*) or Snord67KO-2 cells vs. 4T1 WT cells (*right*), where splicing efficiency = 100% - intron retention rate (%). Each point represents an intron. The black line corresponds to equal splicing efficiency in the KO cells and WT cells. Red points correspond to introns with higher splicing efficiency in KO cells compared to WT cells, and blue points correspond to introns with higher splicing efficiency in WT cells compared to KO cells.

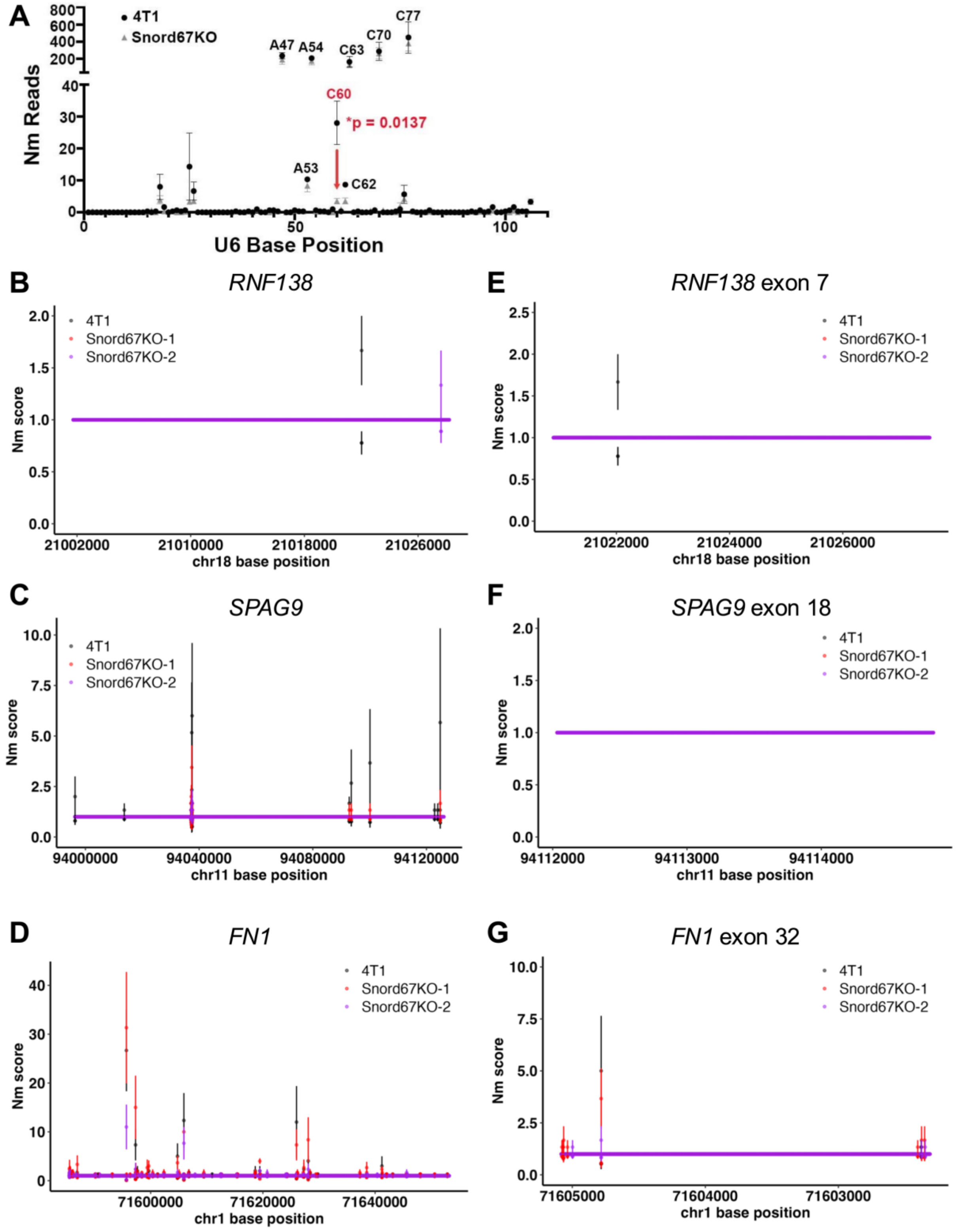

Supplemental Figure 11. Analysis of 2′-*O*-methylation in 4T1 WT cells and Snord67 knockout cells by RibOxi-seq. (**A**) RibOxi-seq mapping of methylation reads in U6 snRNA in

4T1 WT (4T1) and Snord67 knockout (Snord67KO) cells. (**B–G**) RibOxi-Seq mapping of Nm score throughout the whole gene (B–D) and within the alternatively spliced region (E–G) for three genes with cassette exons that exhibit differentially alternative splicing between 4T1 WT cells and two Snord67 knockout clones (Snord67KO-1 and Snord67KO-2). Error bars correspond to ± 1 S.E.

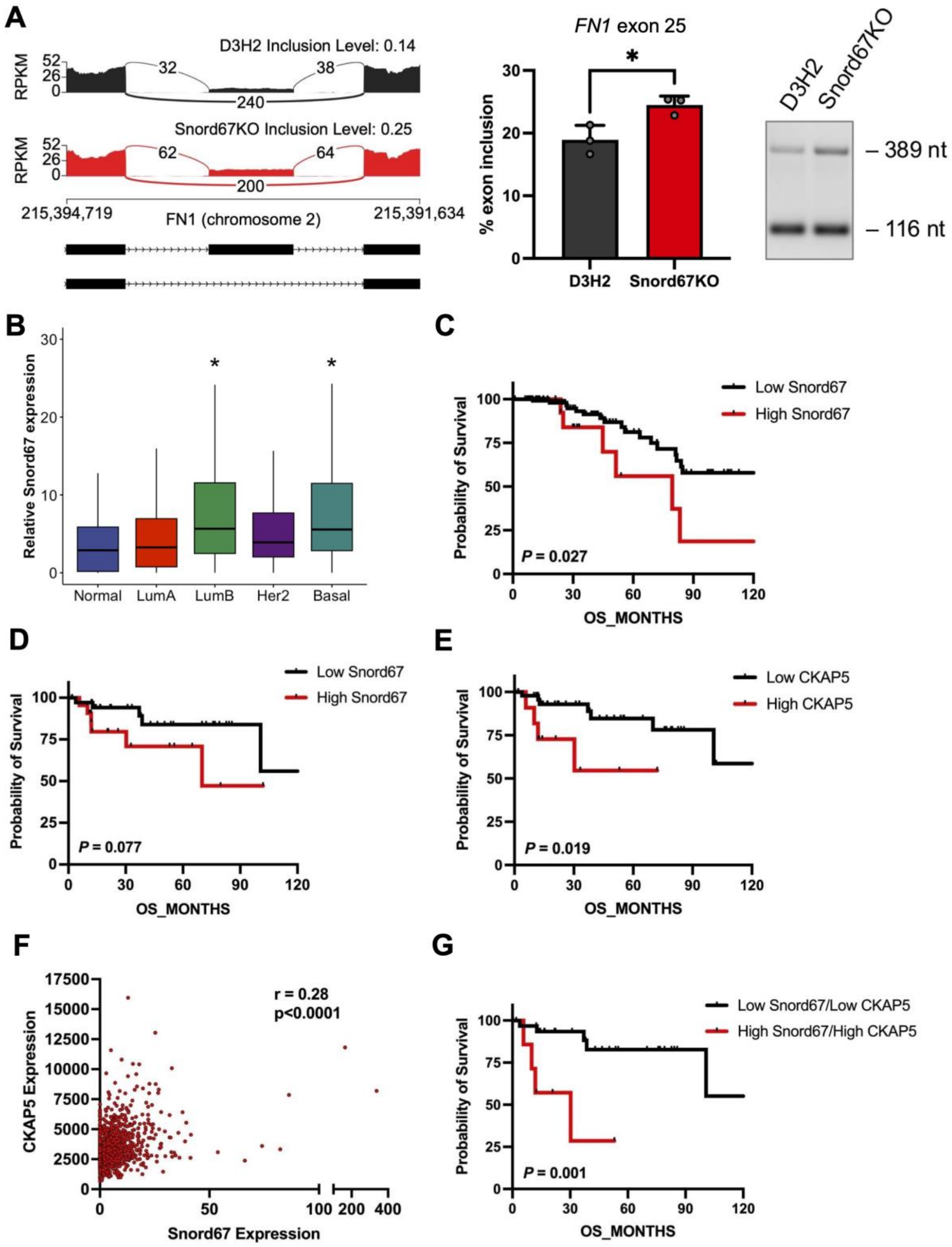

Supplemental Figure 12. Validation of differential alternative splicing in Snord67 knockout vs D3H2 WT and analysis of Snord67 expression in TCGA data. (**A**) *Left*: Sashimi plots for

differential splicing of exon 25 in the *FN1* gene in D3H2 WT cells compared to Snord67KO cells. *Right*: RT–PCR gel, with quantification by densitometry, for validation of differential splicing of exon 25 in the *FN1* gene in D3H2 WT cells compared to Snord67KO cells. The RNA-seq and RT–PCR experiments were performed in n=3 biological replicates. Error bars correspond to +1 S.D. Statistical significance was determined by two-tailed t-test; *$p<0.05$ ($p=0.024$). (**B**) Relative Snord67 expression by breast cancer subtype: normal (n=36), luminal A (n=495), luminal B (n=191), Her2 (n=77), and basal (n=165). Statistical significance was determined by two-tailed t-test, using the Benjamini-Hochberg method to correct for multiple testing; *FDR-adjusted *p*-value <0.05 (adjusted $p=0.0061$ for LumB vs. LumA, 0.029 for Basal vs. LumA). (**C**) Overall survival by Snord67 expression in patients with the luminal B subtype of breast cancer. (**D**) Overall survival by Snord67 expression in patients with the HER2-enriched subtype of breast cancer. (**E**) Overall survival by *CKAP5* expression in patients with the HER2-enriched subtype of breast cancer. (**F**) Pearson correlation between Snord67 and *CKAP5* expression in patients with breast cancer (all subtypes). (**G**) Overall survival with high Snord67 and high *CKAP5* expression compared to low Snord67 and low *CKAP5* expression in patients with the HER2-enriched subtype of breast cancer. For survival analyses, statistical significance was determined by log-rank test.

Gene Symbol	Gene Description	Ratio AxLN/MFP	Ratio AxLN-LuM/AxLN
2810008D09Rik	RIKEN cDNA 2810008D09 gene	1.68226	0.64855
Gm23119	predicted gene, 23119	1.48750	0.71018
Scarna17	small Cajal body-specific RNA 17	1.56472	0.68108
2410006H16Rik	RIKEN cDNA 2410006H16 gene	2.44254	0.44586
Gm22422	predicted gene, 22422	1.80781	0.57619
Il10rb	interleukin 10 receptor, beta	1.40046	0.64862
Neat1	nuclear paraspeckle assembly transcript 1 (non-protein coding)	2.40555	0.40762
Snord1b	small nucleolar RNA, C/D box 1B	1.49123	0.64902
Snord1c	small nucleolar RNA, C/D box 1C	1.54840	0.64509
Snord1c	small nucleolar RNA, C/D box 1C	1.54923	0.63556
Snord104	small nucleolar RNA, C/D box 104	1.53706	0.67291
Snora34	small nucleolar RNA, H/ACA box 34	1.44901	0.68701
Gm25835	predicted gene, 25835	1.54222	0.63436
Snora16a	small nucleolar RNA, H/ACA box 16A	1.40703	0.67232
Snord111	small nucleolar RNA, C/D box 111	1.73464	0.47076
Snord67	small nucleolar RNA, C/D box 67	1.65565	0.56170
Snord90	small nucleolar RNA, C/D box 90	2.10628	0.47847
Gm26079	predicted gene, 26079	1.93116	0.58682
Snord61	small nucleolar RNA, C/D box 61	2.07915	0.47321
Gm25970	predicted gene, 25970	1.71851	0.54023
Snora28	small nucleolar RNA, H/ACA box 28	1.51386	0.65564
Gm22858	predicted gene, 22858	1.61493	0.57474
Snord16a	small nucleolar RNA, C/D box 16A	1.34579	0.71307
Scarna9	small Cajal body-specific RNA 9	1.59294	0.60752
Gm22009	predicted gene, 22009	1.20591	0.75911
5430416N02Rik	RIKEN cDNA 5430416N02 gene	2.11608	0.52848
5430416N02Rik	RIKEN cDNA 5430416N02 gene	2.25268	0.52391
Gm20412	predicted gene 20412	0.94989	0.38742
4930455G09Rik	RIKEN cDNA 4930455G09 gene	1.24982	0.32465
H19	H19, imprinted maternally expressed transcript	2.42053	0.19068

Supplemental Table 1. Noncoding and poorly characterized RNAs that are differentially expressed in AxLN tumors: comparisons of gene expression in AxLN vs. MFP tumors and in AxLN-derived lung metastases (AxLN-LuM) vs. AxLN tumors. Each row designates one microarray probe set. The genes are listed in the same order as they appear on the microarray heat map in Figure 1A. Note that in some cases the microarray included more than one probe set for the same gene.

NEB miRNA linker	5′-/rApp/CTGTAGGCACCATCAAT/NH2/- 3′
RibOxi RT Primer	5′-GTGACTGGAGTTCAGACGTGTGCTCTTCCGATCTNNNNNNATTGATGGTGCCTACAG-3′
5'RNA linker	5′-/Biosg/ACACUCUUUCCCUACACGACGCUCUUCCGAUCUNNNNN-3′
PCR_i5	5′-aatgatacggcgaccaccgagatctacac**-i5(8nt)-**acactctttccctacacgacgctcttccgatct-3′
PCR_i7	5′-caagcagaagacggcatacgagat**-i7(8nt)-**gtgactggagttcagacgtgtgctcttccgatct-3′

Supplemental Table 2. Oligonucleotides used for RNA- and RibOxi-sequencing in 4T1
