# Supplementary material for: Snord67 promotes breast cancer metastasis by guiding U6 modification and modulating the splicing landscape
Source: Nat Commun. 2025 May 2;16:4118. doi: 10.1038/s41467-025-59406-w (PMC12048515; doi:10.1038/s41467-025-59406-w)
Supplement: Supplementary file 2 — Description of Additional Supplementary Files [file 41467_2025_59406_MOESM2_ESM.pdf]

Description of Additional Supplementary Files

Supplementary Data 1. Noncoding and poorly characterized RNAs that are differentially expressed in AxLN tumors compared to MFP tumors. Each row designates one microarray probe set. The genes are listed in the same order as they appear on the microarray heat map in Supplemental Figure 1D. Note that in some cases the microarray included more than one probe set for the same gene.

Supplementary Data 2. Noncoding and poorly characterized RNAs that are differentially expressed in AxLN-derived lung metastases (AxLN-LuM) compared to AxLN tumors. Each row designates one microarray probe set. The genes are listed in the same order as they appear on the microarray heat map in Supplemental Figure 1E. Note that in some cases the microarray included more than one probe set for the same gene.

Supplementary Data 3. Sequences of Primers, CRISPR sgRNAs, and ASOs

Supplementary Data 4. Individual p-values for statistical analyses in main figures not included in the figure legends.
